# Preparation and Performance Evaluation of High-Temperature Resistant Acrylamide/Vinylpyrrolidone Copolymer-Based Gel System

**DOI:** 10.3390/polym18040530

**Published:** 2026-02-21

**Authors:** Zhande Yang, Hua Li, Xiaodong Cao, Hao Wang, Jing Bai, Bowen Chen, Zezhou Fang

**Affiliations:** 1 Xi’an Changqing Chemical Group Co., Ltd., Xi’an 710200, China; 2 Plant No. 8, Changqing Oilfield, Yan’an 710018, China; 3 Shale Gas Research Institute, PetroChina Southwest Oil & Gas Field Company, Chengdu 610051, China; chenbw_2023@petrochina.com.cn; 4 State Key Laboratory of Oil and Gas Reservoir Geology and Exploitation, Southwest Petroleum University, Chengdu 610500, China

**Keywords:** high-temperature polymer gel, plugging property, thermal stability mechanism, enhanced oil recovery, high temperature reservoir

## Abstract

Polymer gels are widely used for profile control and water shutoff in mature reservoirs, while conventional gels are limited under high temperature due to poor thermal stability. This study develops a high-temperature-resistant gel based on acrylamide/vinylpyrrolidone copolymer (P(AM/NVP)), crosslinked with hydroquinone-hexamethylenetetramine (HQ-HMTA). At 150 °C, the gel achieves a Sydansk strength code of H with a gelation time of 9.5 h, and shows excellent thermal stability, maintaining over 90% weight after 180 days. Rheological and microscopic analyses confirm a dense, stable network with high storage modulus (G′). Core flooding tests demonstrate good injectivity with resistance factors of 3.99~129.93, while the plugging rate exceeds 98%. All the experimental results indicate that the P(AM/NVP)-based gel has great potential for water plugging in high temperature oil reservoirs.

## 1. Introduction

Waterflooding is a broadly implemented secondary recovery method in naturally depleted oil reservoirs [1,2,3]. For most types of oil reservoirs, waterflooding is recognized as the most effective and economical way to maintain formation pressure and further improve oil production [4,5]. However, waterflooding recovery is only 30~50% original oil in place (OOIP) to date due to poor displacing and/or sweep efficiency [6,7,8]. Especially for oil reservoirs with high permeability channels or fractures, early water-breakthrough is a serious problem faced by the oil industry as it leads to relatively low oil recovery and the excessive production of water [9,10,11]. The associated operational and economic problems in treating excessively produced water also pose a challenge to the effectiveness of waterflooding [12]. Therefore, water management so as to achieve conformance control and improve the economic viability of waterflooding has been an important subject for the industry.

Polymer gel has been the most prevalent water-shut off agent in mature oil reservoirs in the past decades due to its many benefits, for example, controllable gelation behaviors, easy preparation, and cost-effectiveness [13,14]. When low-viscosity gel base solution is injected into the reservoir through surface pumping or coiled tubing system, polymer gel with three-dimensional network structures is expected to form at the desired zone after aging, which substantially reduces reservoir permeability therein and diverts the subsequently injected water into less-swept (or low permeability) regions [15]. Therefore, polymer gel can effectively plug thief-zones and expand sweep efficiency. The majority of polymer gel systems contain natural or synthetic polymers, such as xanthan gum, partially hydrolyzed polyacrylamide (HPAM) and acrylamide-based copolymer [16,17], as well as crosslinkers and other additives. However, research has demonstrated that xanthan gum and HPAM are inapplicable at elevated temperatures [18,19,20]. Hence, acrylamide-based polymer containing high levels of temperature-resistant side groups (NVP or AMPS) may fulfill the criteria of reservoir temperature above 120 °C [21,22].

As one of the most important gel components, crosslinkers are usually categorized into inorganic and organic types [23]. For inorganic crosslinked gels, ionic bond or coordination bond forms between the negatively charged carboxylate groups on the polymer chain and the polyvalent cation of inorganic crosslinker [24,25]. In the literature, the broad implementation of trivalent cations chromium (Cr^3+^) and aluminum (Al^3+^) as inorganic crosslinkers to form gels with PAM or acrylamide-based copolymer has been reported [24,26,27]. However, syneresis or over cross-linking caused by inorganic crosslinkers may lead to contracted gel structure, thereby degrading gel strength at high temperatures [28,29,30]. For organic crosslinked gel systems, the amide groups on polymer chain are crosslinked by organic crosslinkers—such as phenolic resin—and a covalent bond is formed [31]. Since a covalent bond is more rigid than an ionic bond or coordination bond formed by an inorganic crosslinker, the organic crosslinker has gained more attention for use at elevated temperatures. Organic crosslinkers are composed of phenolic crosslinkers, such as phenol and their derivatives resorcinol (RQ) and hydroquinone (HQ), as well as formaldehyde and less-toxic hexamethylenetetramine (HMTA) [32,33]. It is reported that the “secondary cross-linking” method between phenolic crosslinker and formaldehyde or HMTA can further improve gel strength and water resistance in highly permeable zones [34]. Hutchins et al. [35] synthesized an organic polymer gel system with PAM and HQ-HMTA as crosslinker. The gel system showed ultra-thermal stability at 149 °C for 12 months and 176.7 °C for 5 months, which is applicable to water plugging in highly permeable regions. Baisali Sengupta et al. [12] evaluated the gelation performance of a PAM-HQ-HMTA organic gel. It was found that PAM-HQ-HMTA gel demonstrated high mechanical strength at 120 °C and was inexpensive, which means it can be used as an efficient water shut-off agent to mitigate water production at high temperatures. Yadav et al. [36] investigated the effect of the composition and concentration of polymer and crosslinker, temperature, pH, and salinity on the gelation performance of PAM-HQ-HMTA gel. Liu et al. [37] evaluated the effect of polymer and crosslinker concentration, temperature and pH on PAM-HQ-HMTA gel system. They concluded that 0.6 wt% PAM + 0.6 wt% HQ-HMTA can form a hydrogel with good thermal stability and inferred its application in reservoirs at ultra-high temperature (140 °C) according to DSC analysis. In addition to HQ, the implementation of the RQ-HMTA crosslinking system at a high temperature has also been reported. Sun et al. [2] investigated the bulk gelation performance of polymer gels prepared with RQ-HMTA as a crosslinker and optimized the proportions of RQ-HMTA in the ratio of 1:4. They found that RQ-HMTA crosslinked polymer gel demonstrated benign gel strength and thermal stability. According to the reported research, it is known that with proper selection of crosslinker type and concentration, gelation performance and thermal stability of organic polymer gel can be managed and enhanced [38].

In this study based on the excellent thermal stability of P(AM/NVP), the type and concentration of crosslinker are optimized according to the gelation time and gel strength at 150 °C. Then, rheological properties are characterized by a rheometer, and the microstructure is investigated using a Scanning Electron Microscope (SEM). A TGA test is conducted to investigate thermal stability and corresponding mechanism. Moreover, core plugging experiments are conducted on homogenous and artificial fractured cores to investigate the injectivity of gel base solution and plugging properties of the optimized gel.

## 2. Experimental Section

### 2.1. Materials

The acrylamide-based copolymer P(AM/NVP) (7:3 mole ratio of AM to NVP) with 93% solid content and molecular weight of 6.5 × 10^6^ g/mol used in this study was synthesized via facile water-free-radical polymerization technique in the laboratory according to the literature [39]. Phenol, hydroquinone (HQ), resorcinol (RQ), formaldehyde, hexamethylenetetramine (HMTA), and gel stabilizer (thiourea) were purchased from Kelong Chemical Reagent Co., Ltd. (Chengdu, China). The formaldehyde was used as solution with 37% effective content. All chemical agents were of AR grade. Deionized (DI) water was also prepared in the laboratory.

### 2.2. Preparation and Characterization

#### 2.2.1. Gel Preparation and Gelation Behavior Characterization

1 g P(AM/NVP) was added to a beaker with 100 mL DI water and stirred for more than 2 h until copolymer powder were fully dissolved. Then, different types or dosage of organic crosslinkers and 0.05 g thiourea were added into the beaker and continuously stirred for 20 min to secure complete dissolution. To ensure the same number of benzene rings provided by different phenolic crosslinkers, the concentration of phenol, RQ and HQ were determined to be 0.26 wt%, 0.30 wt%, and 0.30 wt%, respectively. In addition, as HMTA could generate formaldehyde reacting with water at a mole ratio of 1:6, the concentrations of HMTA and formaldehyde were thus determined to be 0.30 wt% and 1.00 wt%, respectively [40].

**Figure 1:**
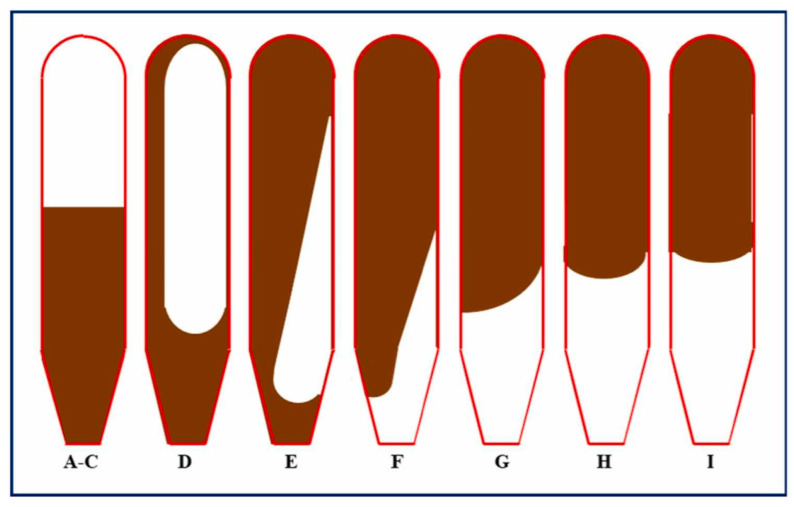
Illustration of Sydansk’s gel strength code [42].

The prepared gel base solutions were poured into temperature- and pressure-resistant glass bottles and placed in a heat oven at 150 °C for aging. During the gelation process, the bottles containing gelant solutions were inverted at different time intervals and the gelation time was recorded. The dehydration percentage and long-term thermal stability were summarized. The gel strength was evaluated using Sydansk’s gel strength code as shown in Figure 1 [41]. An alphabetic code of A through I was used to characterize gel strength wherein A–E represent flowing gels, F–H refer to non-flowing gels and I signify rigid gels [17]. Here, gelation time was determined as the corresponding time intervals between the start of aging and the time once the gel reached the maximum strength. Subsequently, the gelation performance of aged gels was evaluated after aging 180 d.

#### 2.2.2. Viscoelasticity Measurements

Viscoelasticity reflects the strength of the gel’s three-dimensional network structure, which fundamentally determines its mechanical stability and erosion resistance in porous media. Storage modulus (G′) and loss modulus (G″) were measured by a rheometer (MCR302, Anton Paar, Graz, Austria) equipped with a high-pressure and high-temperature cell. The scanning shear frequency varies from 0.1 Hz to 10 Hz at a constant shear mode with a fixed shear stress of 1%.

#### 2.2.3. SEM Analysis

A scanning electron microscope (Quanta 450, FEI, Hillsboro, OR, USA) was deployed to characterize the microstructure of polymer gels [42]. Before taking the micrographs of gels, the gel samples were freeze-dried and gold-sprayed for better observation.

#### 2.2.4. TGA Analysis

A TGA test was carried out to analyze the thermal stability of polymer gels by using a thermogravimetric analyzer (TG 209 F3 Tarsus, Naichi, Selb, Germany). First, a small piece (about 5 mg) of freeze-dried gel sample was cut out for use. Then, the gel sample was placed in an alumina crucible. A nitrogen atmosphere with a flow rate of 20 mL/min was adopted. The experimental temperature range was 30~600 °C and the heating rate was 12.22 °C/min.

### 2.3. Core Plugging Experiments

**Figure 2:**
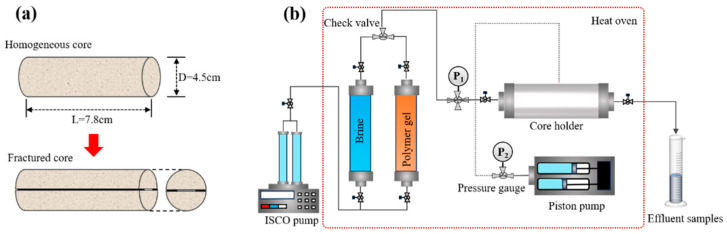
Schematic diagram of fractured-core preparation (**a**) and core plugging tests (**b**).

Five homogenous cylindrical cores with different permeability (283 mD, 405 mD, 983 mD, 1485 mD, 2365 mD) were used to evaluate the injecting performance of the gelant solution. Each cylindrical core was nearly 7.8 cm in length (L) and 3.8 cm (*D*) in diameter. To manufacture fractured core, the homogeneous core was split in two in the middle along the axial direction, and two polytetrafluoroethylene (PTFE) sheets were placed between the two half-cores to generate fractures of different widths (0.3 mm, 0.5 mm, 1.0 mm). Then, the half-cores with PTFE sheets were tightly stuffed within a plastic sealing tube after heating by a heat dryer. The schematic diagram of fractured core preparation and core flow test was shown in Figure 2. The experimental procedure was as follows:

(1) Saturate dried homogeneous core with brine after vacuuming, and calculate pore volume and porosity through mass balance.
(2) Inject brine into the core at 0.3 mL/min, record the pressure difference ($\Delta P_{W}$) between inlet and outlet of the core-holder and calculate matrix permeability ($K_{0}$) according to Darcy’s law.
(3) Inject gelant solution into the core at 0.3 mL/min, record the pressure difference ($\Delta P_{G}$) and calculate resistance factor ($R_{F}$) according to Equation (1).
(4) Initial permeability of fractured core ($K_{a}$) before plugging is calculated according to step (1) and (2).
(5) Put the fracture core with 1 FV (fracture volume) gelant solution into oven at 150 °C for aging for 5 d to obtain complete gelation.
(6) Inject brine into the fractured core at 0.3 mL/min, record breakthrough pressure ($P_{b}$) and stable pressure difference, then calculate permeability of fractured core with gel ($K_{b}$), breakthrough pressure gradient ($P_{L}$) and plugging rate ($\eta_{s}$) according to Equation (2) and Equation (3), respectively.
(7) After plugging experiment, remove the PTFE sheets, measure the matrix permeability ($K^{\prime }$) of core and calculate damage rate ($\eta_{h}$) to matrix permeability.
  (1) $$R_{F}=\frac{\Delta P_{G}}{\Delta P_{W}}$$
  (2) $$P_{L}=\frac{P_{b}}{L}$$
  (3) $$\eta_{s}=\left(1-\frac{K_{b}}{K_{a}}\right)\times 100\%$$
  (4) $$\eta_{h}=\left(1-\frac{K^{\prime }}{K_{0}}\right)\times 100\%$$

## 3. Results and Discussion

### 3.1. Gelation Performance

#### 3.1.1. Screening of High-Temperature Crosslinker Type

The gel performance of polymer gels with various crosslinker types before and after aging is shown in Table 1. It can be seen that HMTA can significantly increase gel performance, such as prolonged gelation time and greater gel strength compared with formaldehyde at the same test conditions. This is largely because that HMTA slowly decomposes formaldehyde and ammonia gas at an elevated temperature and acid condition, thus decreasing the crosslinking rate at high temperatures [43]. The underlying mechanism stems from a shift in the decomposition pathway—changing from a localized, controlled decomposition under ideal conditions to a bulk, explosive homogeneous hydrolysis. This leads to the volatilization loss of formaldehyde, the escape of ammonia gas causing catalytic imbalance, and the failure to effectively concentrate reactive intermediates at polymer sites. Ultimately, this results in an insufficient supply of cross-linking reactants and defects in network formation, thereby reducing cross-linking efficiency. The gelation time of phenol-formaldehyde crosslinked gel is 5.5 h, and the gel strength code approaches level G and decreases to level F after aging. When HMTA substitutes the role of formaldehyde, the gelation time increases to 7.5 h with gel strength increasing to code H. HQ demonstrates better gelation performance and thermal stability whether co-crosslinking with formaldehyde or HMTA compared to other phenolic crosslinkers. The corresponding gelation time of HQ-formaldehyde and HQ-HMTA is 7.5 h and 9.5 h, and the gel strength reaches code G and code H, respectively. Meanwhile, HQ-formaldehyde and HQ-HMTA crosslinked gels remain stable in 150 °C after aging. However, the gelation time of the RQ-formaldehyde crosslinked gel is only 2.5 h and the gel strength is code G before and after aging. The gelation time of the RQ-HMTA crosslinked gel increases to 5.5 h with the same level of gel strength as RQ-formaldehyde gel. The relatively short gelation time of RQ-formaldehyde and RQ-HMTA gel is caused by the phenolic hydroxyl groups in the ortho positions of RQ, which provides more active sites to react with formaldehyde to form low-molecular-weight phenolic resin crosslinkers compared to phenol and HQ [44]. The RQ-formaldehyde system exhibits the fastest gelation because resorcinol acts as a highly reactive autocatalyst and synergizes with readily available free formaldehyde, substantially accelerating the condensation reaction. In contrast, the HQ-HMTA system shows the slowest gelation, as its formaldehyde must be slowly released through the thermal decomposition of HMTA, and hydroquinone further inhibits the reaction process by acting as a radical scavenger, resulting in a significant difference in gelation times [45]. The gelation performance of the organic gels after aging is shown in Figure S1.

#### 3.1.2. Screening of Crosslinker Concentration

In order to develop a gel system with optimized gel performance, the effect of HMTA concentration on gel strength and gelation time under the condition of HQ concentration is 0.3% and the results were tabulated in Table 2. The gelation time of HQ-HMTA gels decreases from 20 h to 4 h with increasing HMTA concentrations from 0.1 wt% to 0.55 wt%, and the gel strength code increases from level F to H. After the aging process, a dehydration percentage of 10~50% was observed when the mass concentration of HMTA was less than 0.2 wt%. When the mass fraction of HMTA exceeds 0.5 wt%, the dehydration percentage exceeds 10% after aging and the gel strength code decreases to level F due largely to over-crosslinking. Therefore, 0.3 wt% HMTA is identified as the optimal concentration for co-crosslinking with 0.3 wt% HQ, resulting in a stable gel with a relatively long gelation time of 9.5 h. This is notably longer than the 6 h gelation time observed with 0.4 wt% HMTA. In the following discussions, the gelant solution refers to the solution of 1 wt% P(AM/NVP) + 0.3 wt% HQ + 0.3 wt% HMTA, the corresponded gel is called HQ-HMTA gel.

### 3.2. Viscoelasticity Properties of Gel

**Figure 3:**
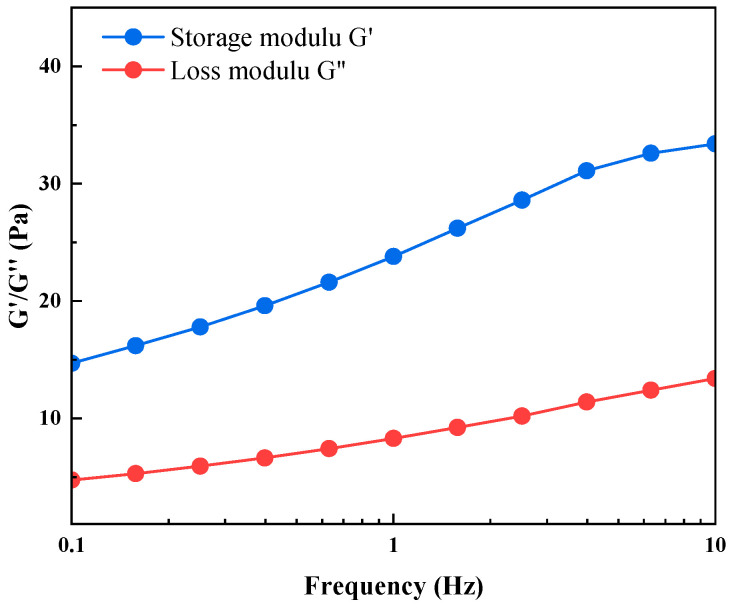
Storage modulus G′ and loss modulus G″ of HQ-HMTA gel as a function of shear frequency.

Viscoelasticity tests were performed to evaluate the optimized HQ-HMTA gel after gelation. Figure 3 shows the viscoelasticity test result of HQ-HMTA gel, both the storage modulus (G′) and loss modulus (G″) increase as the shear frequency increases. The G′ of HQ-HMTA gel is always higher than G″ at different shear frequencies, exhibiting the elastic properties of HQ-HMTA gel and certain level of deforming and restoring abilities. At a shear frequency of 0.1 Hz, the G′ of HQ-HMTA gel is 14.7 Pa and the G″ is higher than 2 Pa, indicating the strong gel strength of the HQ-HMTA gel. The rheology result of other organic gels is shown in Figure S2. It can be seen that HQ-HMTA gel demonstrates higher G′ than other gels, which was ascribed to the unique synergistic effect: HMTA acts as a latent crosslinker that slowly releases formaldehyde through decomposition, while hydroquinone (HQ) provides inhibition/delaying functions. Together, they promote the formation of a more homogeneous three-dimensional network with a moderate crosslinking density. Compared to fast-crosslinking systems, the HQ-HMTA system delays the gelation process, allowing for sufficient chain extension and entanglement. This results in longer effective load-bearing chain segments and a greater number of flexible methylene ether bridges. At the microscopic level, this constructs a gel network that combines high elastic recovery capability with uniform stress distribution, macroscopically manifesting as the simultaneous enhancement of mechanical strength and flexibility.

### 3.3. Microstructure of Gel

**Figure 4:**
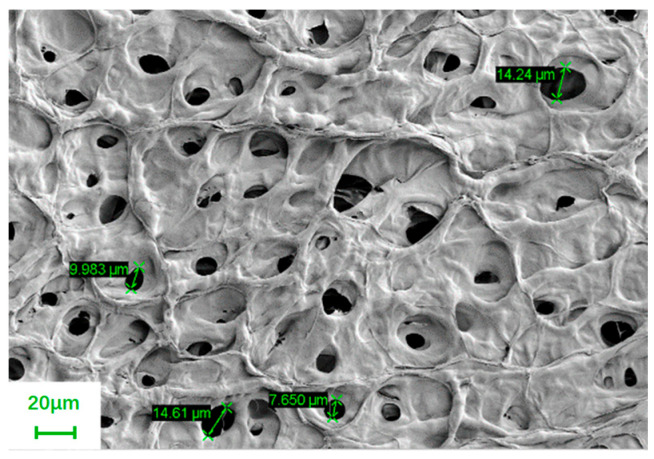
SEM image of HQ-HMTA d gel after gelation.

Microscopic morphology of polymer gels determines its macroscopic properties, such as gel strength and thermal stability. Figure 4 shows the SEM micrograph of HQ-HMTA hydrogel after gelation. It can be seen that the HQ-HMTA gel possess uniformly distributed 3D network structures. The network skeleton of the HQ-HMTA gel is bulky and densely covered with pore-like meshes. The size of those pore-like meshes ranges between 7.6 and 14.2 μm. The density of the gel network is related to the structure and concentration polymers, as well as the type and concentration of crosslinking agents. The gelation experiments in this study were conducted at a single temperature, making it impossible to calculate gelation kinetic parameters. But the SEM result shows that the gel structure with small pore size and large skeleton facilitates the bonding of water in the bulk gel, which contributes to a lower water separation rate and remains more stable and rigid at high temperatures.

### 3.4. Thermal Stability Mechanism of Gel

**Figure 5:**
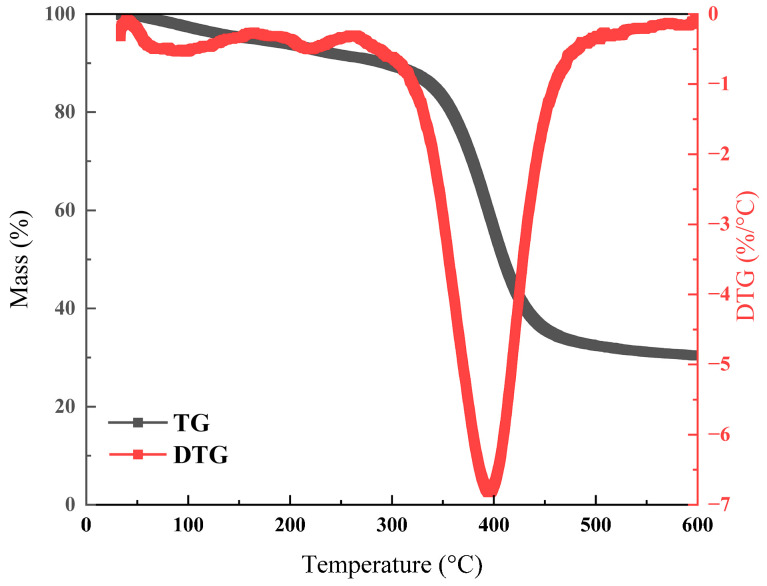
TG/DTG analysis of HQ-HMTA gel after aging.

TG analysis result of HQ-HMTA gel sample is shown in Figure 5. The weight loss condition can be categorized into three heating stages. The weight loss of the gel in the first heating stage (30~150 °C) refers to free and crystalized water evaporation, the second stage (150~300 °C) demonstrates carbonization of crosslinking structures and the third stage (300~600 °C) indicates thermal cracking of polymer chains. As shown in Figure 5, the theoretical residue of HQ-HMTA gel is 33.4% after heating treatment up to 600 °C. At the second and third heating stage, nearly 20% and 50% weight loss of the gel are observed from TG thermogram, respectively. The peak temperature of 212 °C is observed in the second stage and the maximized mass weight loss occurred at 389 °C from DTG curve, indicating good thermal stability of HQ-HMTA gel at 150 °C. However, as the structures of polymers or crosslinking agents vary across the literature, the degradation of functional groups may be different. In subsequent research, TG-FTIR coupling characterization is recommended to analyze the degradation of functional groups at different temperatures. Moreover, TGA only simulates the effect of temperature on gel stability, while long-term stability experiments of gels take into account the influence of temperature on stability under aqueous conditions. Therefore, thermogravimetric experiments serve as a supplementary approach to assessing the long-term stability of gels.

**Figure 6:**
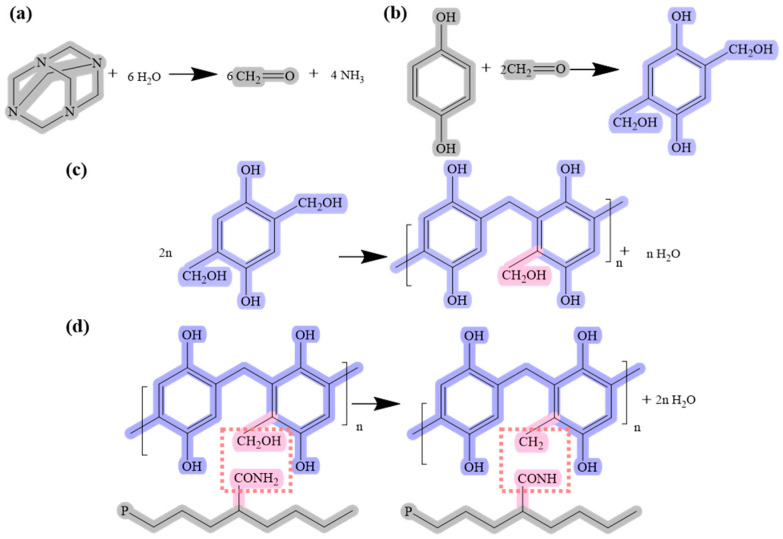
Gelation mechanism of HQ-HMTA and P(AM/NVP).

The gelation mechanism of P-HQ-HMTA is shown in Figure 6. HMTA decomposes formaldehyde and NH_3_ at high temperatures, and formaldehyde reacts with HQ to form a 2,5-hydroxymethyl phenol. Subsequently, multiple 2,5-hydroxymethyl phenol are condensed to form phenolic resins with the release of water, and cross-linked into a cluster gel structure. A 3D network gel can be formed through condensation reactions between the hydroxyl groups of phenolic resins and the amine groups on the polymer chain, thus a higher bond energy and better thermal stability is obtained.

### 3.5. Plugging Performance of Gel

#### 3.5.1. Injectivity of Gel in Homogenous Cores

**Figure 7:**
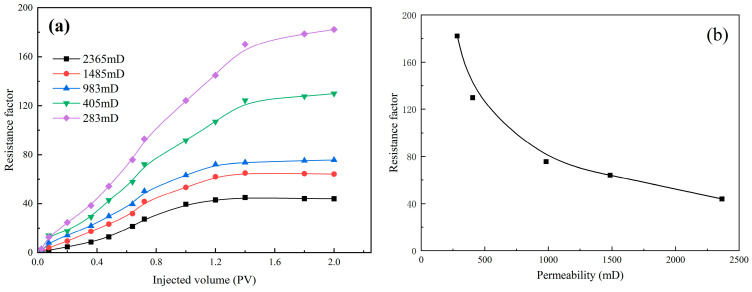
Resistance factor of HQ-HMTA gelant solution in different homogenous cores against injected volumes: (**a**) change in resistance factor during injection process, (**b**) relationship between permeability and resistance factor.

The injectivity of the gelant solution has a great impact on the application of in situ formed polymer gel. Figure 7 shows the resistance factor of HQ-HMTA gelant solution in different homogenous cores against injected pore volumes. It can be seen that the resistance factor increases as the injected volume increases, and a lower resistance factor is obtained in high permeability cores. In an homogenous core with a permeability above 900 mD, the resistance factor increases with an increasing injection volume of gelant solution before reaching a stable level at 1.2~1.6 PV, while the resistance factor continues increasing before reaching a stable level at 1.8~2.0 PV for cores with a lower permeability than 900 mD. The resistance factor value varies from 3.99 to 129.93, showing better injectivity of the optimal HQ-HMTA gelant solution. In addition, the polymers used in this study are specified, including their structures and molecular weights. In future work, the influence of polymer type and molecular weight on injectability will be investigated.

#### 3.5.2. Plugging Effect of Gel on Fractured Cores

**Figure 8:**
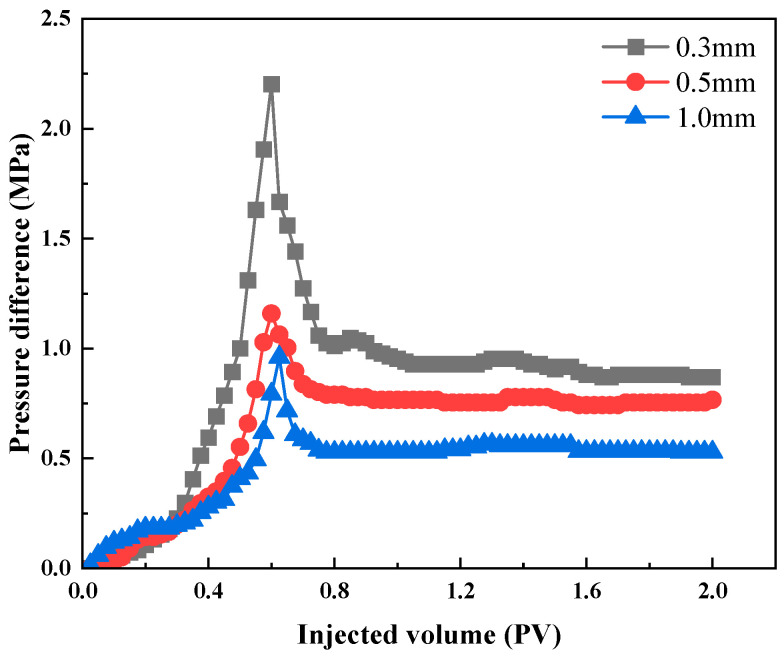
Pressure difference in gel-plugged fractured core of different fracture length.

The fractures in artificial fractured cores are regarded as highly permeable flowing channels. As shown in Table 3, the plugging rate slightly decreases with increase in fracture width. As the fracture width increases from 0.3 mm to 1 mm, the breakthrough pressure gradient decreases from 24.32 MPa/m to 12.28 MPa/m, yet the overall plugging rate is above 98%, indicating good plugging performance and a certain level of adhesion of HQ-HMTA gel at high temperatures. Figure 8 shows the pressure difference curve in fractured cores of different fracture length. To elucidate the damage effect of HQ-HMTA gel on matrix pores, matrix permeability before and after plugging is calculated as shown in Table 4. It can be seen that matrix permeability changes slightly, indicating relatively low damage for matrix pores caused by the injection of HQ-HMTA gel. Moreover, the damage on the matrix pores is relatively higher for cores with higher permeabilities.

## 4. Conclusions

This study advances the molecular design of high-temperature conformance control agents by establishing a synergistic crosslinking strategy and elucidating the underlying structure–property–performance relationships. The principal scientific contributions are:

Synergistic crosslinking mechanism for extreme conditions: We demonstrate that the HQ-HMTA system operates through a complementary mechanism, where HMTA functions as a kinetic moderator to delay network formation, while HQ ensures robust final crosslinking. This synergy enables precise control over gelation kinetics and yields a network with superior architectural stability at 150 °C, overcoming the limitations of conventional single crosslinkers.

Quantitative microstructure-property correlation: A definitive causal link is established between nanoscale architecture and macroscopic performance. The gel’s dominant elastic response (G′ > G″), exceptional long-term thermal stability (minimal syneresis after 180 days), and high thermal degradation threshold are mechanistically attributed to its continuous, densely crosslinked three-dimensional network with homogeneous pore-size distribution.

Validated engineering performance in heterogeneous media: The engineered gel demonstrates functionally discrete behavior in porous media: it maintains deep injectivity and formation compatibility in matrix rock while forming a rigid, impermeable seal in fractured zones (>98% plugging efficiency). This proves its dual capability for in-depth fluid diversion and high-contrast shutoff—critical for managing complex high-temperature reservoirs.

These findings provide a generalizable framework for designing next-generation polymer gels, shifting the paradigm from empirical formulation to mechanism-driven optimization for extreme reservoir applications.

## Figures and Tables

**Table 1:** Gelation performance of polymer gels organically crosslinked with different types of crosslinkers.

Crosslinker	Gelation Time (h)	Gel Strength Code	Gel Strength Code After Aging	Dehydration Percentage (%)
Phenol-formaldehyde	5.5	G	F	0
Phenol-HMTA	7.5	H	H	0
RQ-formaldehyde	2.5	F	F	0
RQ-HMTA	5.5	G	G	0
HQ-formaldehyde	7.5	G	G	0
HQ-HMTA	9.5	H	H	0

**Table 2:** Gel strength and gelation time of gels with different HQ-HMTA concentrations.

HMTA Concentration (wt%)	Gelation Time (h)	Gel Strength Code	Gel Strength Code After Aging	Dehydration Rate (%)
0.10	20	F	-	>50
0.20	12	G	G	10
0.30	9.5	H	H	0
0.40	6	H	H	0
0.55	4	H	F	10

**Table 3:** Plugging performance of HQ-HMTA gel in fractured cores.

Fracture Width (mm)	$K_{a}$ (mD)	$K_{b}$ (mD)	$P_{b}$ (MPa)	$P_{L}$ (MPa/m)	$\eta_{s}$ (%)
0.3	4015	27	1.90	24.32	99.33
0.5	7646	90	1.21	15.56	98.82
1.0	10,547	164	0.96	12.28	98.45

**Table 4:** Damage rate of matrix permeability by HQ-HMTA gel in fractured cores.

Fracture Width (mm)	$K_{0}$ (mD)	$K^{\prime }$ (mD)	$\eta_{s}$ (%)
0.5	65	64	1.53
0.5	510	490	3.90
0.5	896	850	4.98

## Data Availability

The original contributions presented in this study are included in the article/Supplementary Material. Further inquiries can be directed to the corresponding author.

## Supplementary Materials

The following supporting information can be downloaded at: https://www.mdpi.com/article/10.3390/polym18040530/s1, Figure S1: Gelation performance of different organic gel after aging 1 h, 2.5 h, 5.5 h, 7.5 h, 9.5 h: (a) phenol/formaldehyde; (b) phenol/HMTA; (c) RQ/formaldehyde; (d) RQ/HMTA; (e) HQ/formaldehyde; (f) HQ/HMTA, Figure S2: Storage modulus G′ (a) and loss modulus G″ (b) of organic gels as a function of shear frequency, Figure S3: SEM images of organic gels after gelation: (a) phenol/formaldehyde; (b) phenol/HMTA; (c) RQ/formaldehyde; (d) RQ/HMTA; (e) HQ/formaldehyde.
