# Supplementary material for: Preparation and Performance Evaluation of High-Temperature Resistant Acrylamide/Vinylpyrrolidone Copolymer-Based Gel System
Source: Polymers (Basel). 2026 Feb 21;18(4):530. doi: 10.3390/polym18040530 (PMC12944307; doi:10.3390/polym18040530)
Supplement: Supplementary file 1 [file polymers-18-00530-s001.zip › polymers-4067742-supplementary.pdf]

Supplementary Materials

3.1 Gelation performance

3.1.1 Screening of high-temperature crosslinker type

Figure S1 shows the images of organic gels crosslinked by different types of crosslinkers after aging 1h~9.5h.

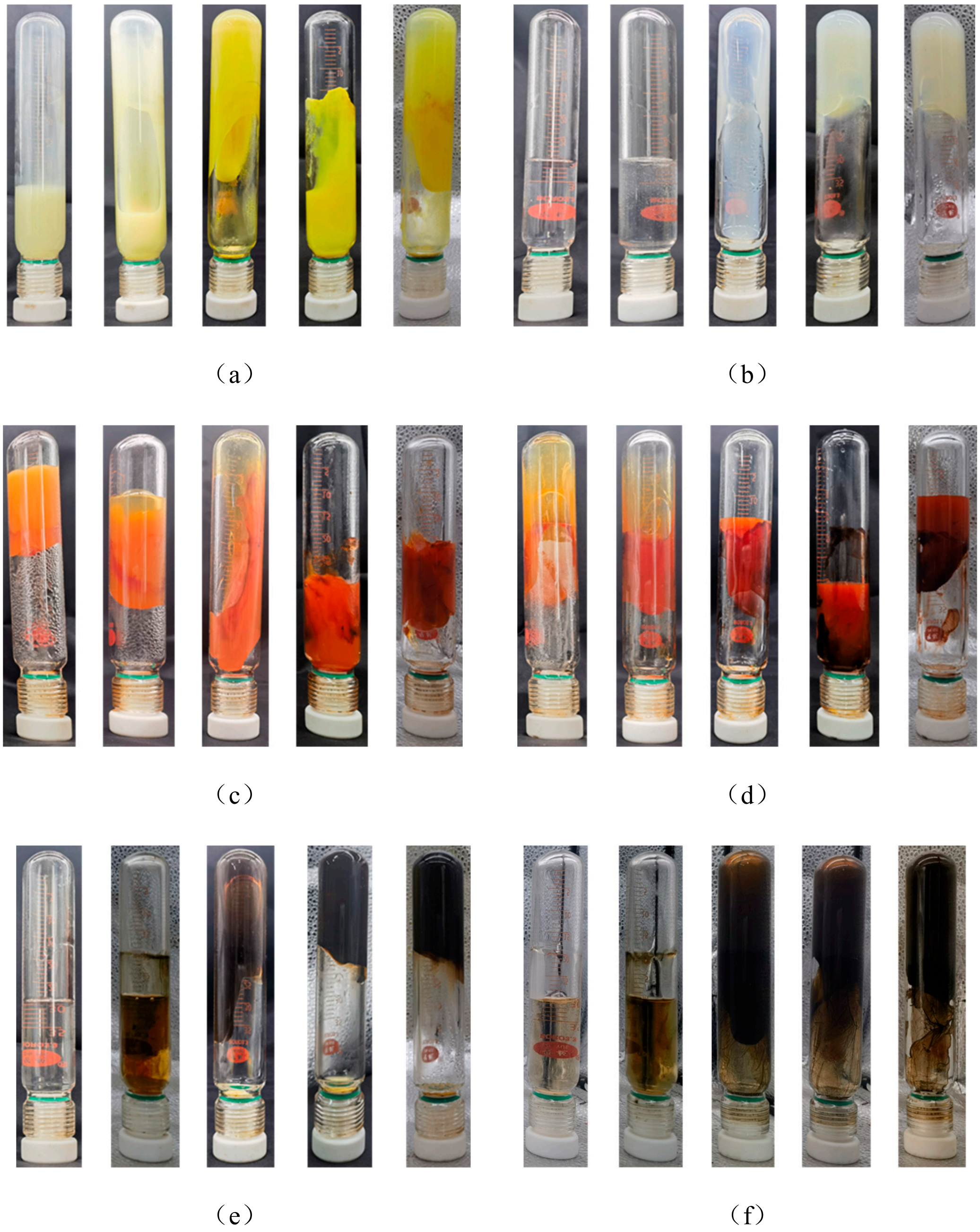

Figure S1 Gelation performance of different organic gel after aging 1h, 2.5h, 5.5h, 7.5h, 9.5h:

(a)phenol/formaldehyde; (b)phenol/HMTA; (c)RQ/formaldehyde; (d)RQ/HMTA; (e) HQ/formaldehyde; (f)HQ/HMTA.

3.2 Rheological properties of gel

As shown in **Figure S2**, the storage modulus G’ and loss modulus G’’ of the gels increase as the shear frequency increases.

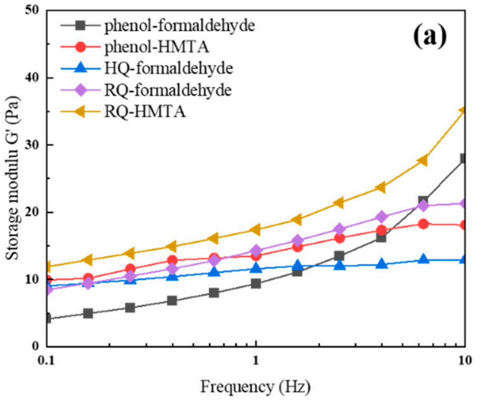

Figure S2 Storage modulus G’ (a) and loss modulus G’’ (b) of organic gels as a function of shear frequency

3.3 Microstructures of gel

Figure S3 shows the microstructure of organic gels after gelation.

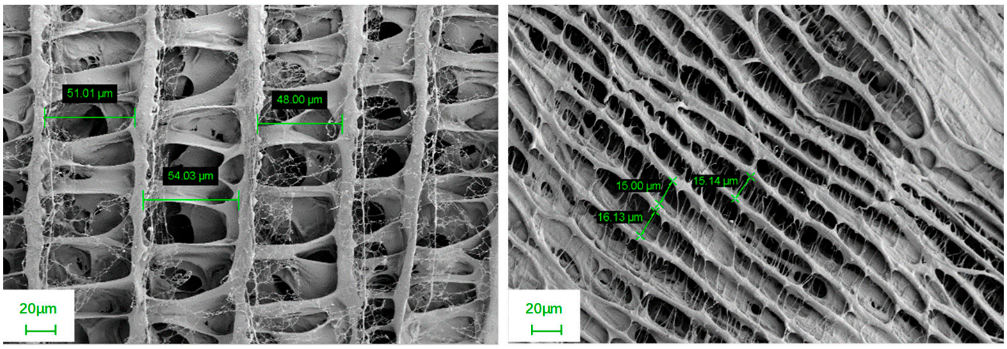

（a） （b）

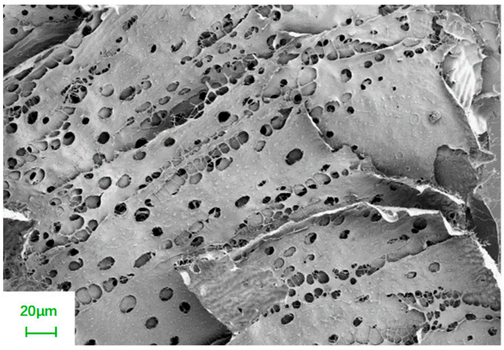

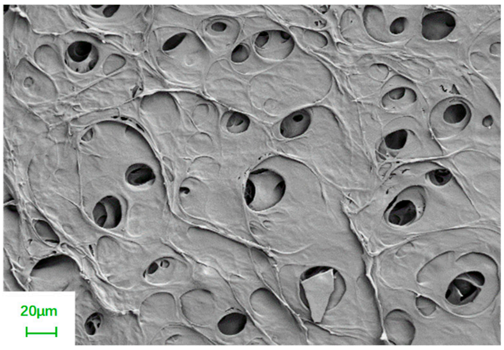

（c）　　　　（d）

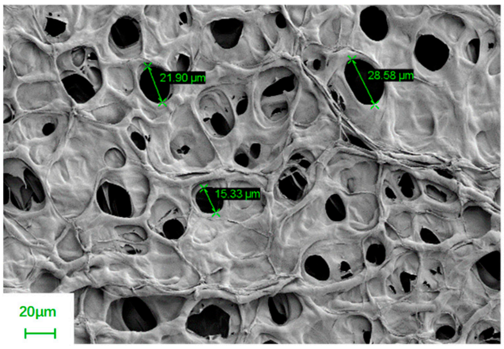

（e）

Figure S3 SEM images of organic gels after gelation: (a)phenol/formaldehyde; (b)phenol/HMTA; (c)RQ/formaldehyde; (d)RQ/HMTA; (e) HQ/formaldehyde.
